# *CenFind*: a deep-learning pipeline for efficient centriole detection in microscopy datasets

**DOI:** 10.1186/s12859-023-05214-2

**Published:** 2023-03-28

**Authors:** Léo Bürgy, Martin Weigert, Georgios Hatzopoulos, Matthias Minder, Adrien Journé, Sahand Jamal Rahi, Pierre Gönczy

**Affiliations:** 1 Swiss Institute for Experimental Cancer Research, School of Life Sciences, Swiss Federal Institute of Technology Lausanne, 1015 Lausanne, Switzerland; 2 Interschool Institute of Bioengineering, School of Life Sciences, Swiss Federal Institute of Technology Lausanne, 1015 Lausanne, Switzerland; 3 Institute of Physics, Swiss Federal Institute of Technology Lausanne, 1015 Lausanne, Switzerland; 4 SBB Consulting, Hilfikerstrasse 1, 3000 Bern 65, Switzerland

**Keywords:** Image analysis, Deep learning, Microscopy, Software, Cell biology

## Abstract

**Background:**

High-throughput and selective detection of organelles in immunofluorescence images is an important but demanding task in cell biology. The centriole organelle is critical for fundamental cellular processes, and its accurate detection is key for analysing centriole function in health and disease. Centriole detection in human tissue culture cells has been achieved typically by manual determination of organelle number per cell. However, manual cell scoring of centrioles has a low throughput and is not reproducible. Published semi-automated methods tally the centrosome surrounding centrioles and not centrioles themselves. Furthermore, such methods rely on hard-coded parameters or require a multichannel input for cross-correlation. Therefore, there is a need for developing an efficient and versatile pipeline for the automatic detection of centrioles in single channel immunofluorescence datasets.

**Results:**

We developed a deep-learning pipeline termed *CenFind* that automatically scores cells for centriole numbers in immunofluorescence images of human cells. *CenFind* relies on the multi-scale convolution neural network *SpotNet*, which allows the accurate detection of sparse and minute foci in high resolution images. We built a dataset using different experimental settings and used it to train the model and evaluate existing detection methods. The resulting average F_1_-score achieved by *CenFind* is > 90% across the test set, demonstrating the robustness of the pipeline. Moreover, using the *StarDist*-based nucleus detector, we link the centrioles and procentrioles detected with *CenFind* to the cell containing them, overall enabling automatic scoring of centriole numbers per cell.

**Conclusions:**

Efficient, accurate, channel-intrinsic and reproducible detection of centrioles is an important unmet need in the field. Existing methods are either not discriminative enough or focus on a fixed multi-channel input. To fill this methodological gap, we developed *CenFind*, a command line interface pipeline that automates cell scoring of centrioles, thereby enabling channel-intrinsic, accurate and reproducible detection across experimental modalities. Moreover, the modular nature of *CenFind* enables its integration in other pipelines. Overall, we anticipate *CenFind* to prove critical for accelerating discoveries in the field.

**Supplementary Information:**

The online version contains supplementary material available at 10.1186/s12859-023-05214-2.

## Background

**Fig. 1:**
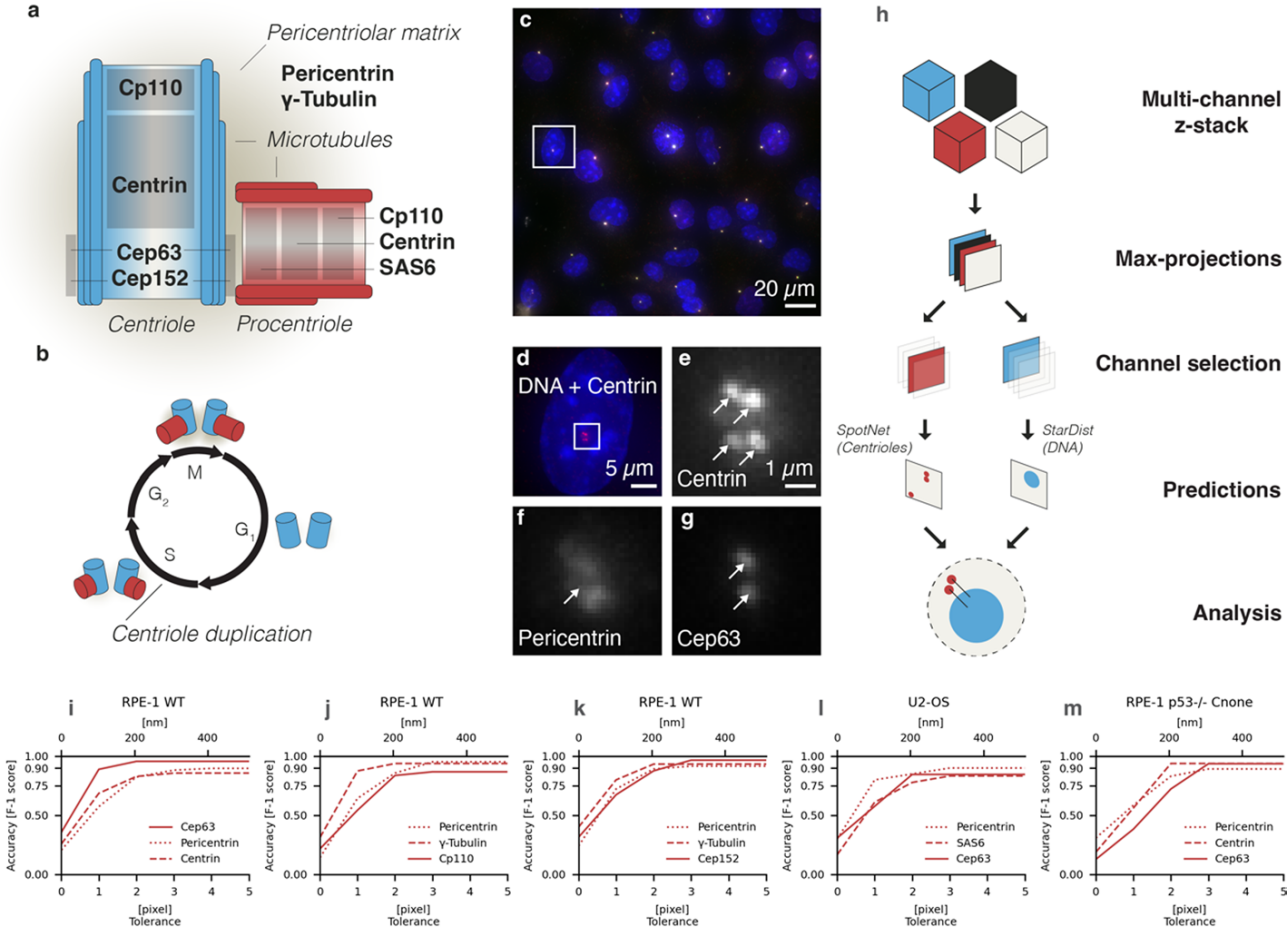
Markers used to detect centrioles, procentrioles and PCM.** a** Schematic representation of centriole (blue) and procentriole (red), with locations of centriole, procentriole and PCM markers used in the dataset.** b** Schematic representation of centriole duplication cycle. M: Mitosis; G_1_ Gap 1 phase; S: synthesis; G_2_: Gap 2 phase.** c**. Representative image of human hTERT-RPE-1 cells immunostained for centrin (red), Cep63 (green), PCNT (cyan) and stained for DNA (blue); box indicates region magnified in **d**–**g**. **d–g** Individual channels: Centrin (**e**), Pericentrin (**f**) and Cep63 (**g**). Arrows indicate foci positions. Note the difference in shape and intensity between centriolar markers.** h** The input for *CenFind* is a multi-channel z-stack, which is max-projected along the z-axis. Channels of interest are selected and passed onto the respective models for centriole/procentriole/PCM detection with *SpotNet* (left) and nucleus segmentation with *StarDist* (right), before saving as coordinates (left) or masks (right). For cell scoring, the nuclei are assigned to centrioles/procentrioles/PCM using a distance metric; the analysis can be carried out using the original image data, together with the saved positions of centrioles/procentriole/PCM. **i–m** F_1_-scores on the test set at different pixel (bottom X axis) and distance (top X axis) tolerances, defined as the distance between an annotation and a prediction. The model needs to yield excellent performance for tolerances as low as 3 pixels to tell apart centriole and procentriole, which are a mere ~ 300 nm away from one another

The centrosome is the principal microtubule organizing centre of animal cells and is typically located in the vicinity of the nucleus (reviewed in [1]). In the S and G2 phases of the cell cycle, the centrosome comprises one centriole ~ 500 nm × 250 nm in dimensions and a neighbouring procentriole, which are both embedded in the pericentriolar matrix (PCM) (Fig. 1a, b). Centrioles are pivotal for fundamental cellular processes, including signalling, motility, and division. Centriole copy number is carefully regulated: most proliferating cells are born with two centrioles, each of which then seeds the formation of a neighbouring procentriole starting approximately at the G1/S transition (Fig. 1b). Understanding the mechanisms governing centriole copy number is an important pursuit in biology with implications for human health, since defects in centriole number and structure can cause disease, including ciliopathies and cancers (reviewed in [2]).

Accurate detection of centrioles in immunofluorescence microscopy experiments is paramount for unravelling centriole copy number mechanisms. Some proteins are present on both centrioles and procentrioles (e.g., Centrin), whereas others mark only centrioles (e.g., Cep63) or only procentrioles (e.g., SAS6) (Fig. 1a, c). Scientists typically use antibodies against such proteins to manually determine centriole/procentriole number per cell, making the process slow and error prone. In principle, automated detection could circumvent these limitations by empirically defining a signal intensity threshold above which pixels are considered as belonging to centrioles/procentrioles. Alternatively, microscopy images can be convolved with a fixed kernel whose weights model the shape of centriolar cylinders. A combination of thresholding and correlating two centriolar markers has enabled the automated detection of centrosome numbers during mitosis [3]. However, such an approach relies on hard-coded parameters, rendering it vulnerable to non-specific immunostaining, changes in signal intensity, as well as variation across cells and experiments.

Deep learning approaches represent an attractive alternative to alleviate these vulnerabilities, and such an approach has been in fact used to detect centrosomes [4]. Although both prior automated approaches use centriolar markers, they served to detect centrosomes, not centrioles and procentrioles *per se*. Moreover, in both cases, detection relies on multiple markers to enhance accuracy. Indeed, detecting centrioles and procentrioles as separate entities is more challenging because they are very close to each other, their distal ends being generally separated by merely ~ 300 nm.

Here, to circumvent the limitations of previous methods, we developed *CenFind*, a robust and fully automated pipeline that scores cells for centriole and procentriole numbers in immunofluorescence images, and this in a channel-intrinsic manner. In the following, we first motivate the design of *CenFind* and then introduce the experimental dataset that we built for evaluation. Thereafter, we describe the overall architecture of *CenFind*, discuss how we trained *SpotNet* to detect centrioles, and then demonstrate the high performance of *CenFind* on our dataset.

### Implementation

We designed *CenFind* to (1) work without manual intervention; (2) process any number of images via a command line interface; (3) function in a channel-intrinsic manner; (4) return the number of centriole and procentriole per cell, using an additional channel for nuclear detection; (5) provide a reusable output for further analysis and retraining. *CenFind* is organized into a data layer, a detection layer, and a scoring layer. The first layer consists of a Dataset class and a Field class, which represent the existing fields of view belonging to one dataset. The second layer contains the centriole detector [5] and the *StarDist*-based nucleus detector [6], as well as the training and evaluation programs. The third layer combines the iteration over fields, detectors, and scoring of cells, namely the assignment of centrioles to nuclei. The user interface consists of command line interface subprograms (prepare, score, evaluate) that perform the different namesake tasks. The *CenFind* pipeline iterates over all the z-stacked or projected fields of view.

To determine the number of centrioles/procentrioles per cell, the pipeline first applies the multi-scale convolutional model *SpotNet* [5] on the channel of interest to detect centriolar and procentriolar foci as single entities provided they are ~ 300 nm apart, which corresponds to 3 pixels in our images (Fig. 1c–g). To determine the number of centrioles or procentrioles per cell, *CenFind* segments the nuclei using the *StarDist* pretrained fluorescence model (Additional file 1: Tables S1 and S2) and assigns the detected centrioles/procentrioles to the closest nucleus (Fig. 1h) [6]. For each field of view, the pipeline runs the detection of centrioles/procentrioles and the segmentation of the nuclei. The marker used for centriole/procentriole detection is set by the user as a function of the experiment. Thereafter, centrioles/procentrioles that are within 3 pixels of one another are assigned jointly to the nearest detected nucleus. In the following, we describe the model *SpotNet* in further details.

*SpotNet* [5] is a multiscale U-Net architecture implemented in TensorFlow 2 using the backbone code from the CSBDeep library [7]. Internally, *SpotNet* first predicts Gaussian probability maps, whereby each spot location is represented by a Gaussian at 3 different resolution levels (Additional file 1: Fig. S1). Specifically, single channel images are tiled into 512 × 512 patches and passed to the model as input layer. In the encoder part, for each of the three levels, the input is convolved twice with a 3 × 3 kernel and then max-pooled with a 4 × 4 kernel, before caching for later up-sampling. In the decoder part, the same steps are carried out in the reverse order and the previously cached layers are concatenated. The model loss is accumulated from the three resolution levels losses and the kernels are initialised using the He method [8]. From the predicted probability maps, the pipeline then extracts the local maxima into a list of predicted centriole/procentriole positions. Importantly, to prevent gradient vanishing when the input is very sparse, the network is allowed to compute down-scaled targets that retain enough signal to provide meaningful gradients.

## Results

### Dataset

The absence of publicly available annotated dataset for evaluating automated centriole/procentriole detection prevents rigorous comparison across models. To fill this gap, we have collected a representative set of images of cells immunostained with various centriole/procentriole/PCM markers. Our five datasets are available at https://doi.org/10.6084/m9.figshare.21581358 and cover two cell types, as well as a condition in which centriole copy number is decreased experimentally (DS1–5; Table 1, Additional file 1: Table S1). Each dataset contains 24 or 25 fields of view imaged for four markers, three labelling centriole/procentriole/PCM plus one labelling nuclei, and is encoded in 16-bit channels. To maximize the generic ability of the pipeline, for each field of view, we segmented all nuclei present in the image and annotated the position of all centrioles based on one marker at random, while ensure an even representation of each marker in the training (~ 90% of the total) and test (~ 10% of the total) sets.

**Table 1:** Markers used in each dataset

Dataset	Cell type	Treatment	Channel 1	Channel 2	Channel 3
DS1	RPE-1	–	Cep63 (C)	Centrin-2 (C, P)	Pericentrin (M)
DS2	RPE-1	–	CP110 (C, P)	γ-tubulin (C, P)	Pericentrin (M)
DS3	RPE-1	–	Cep152 (C)	γ-tubulin (C, P)	Pericentrin (M)
DS4	U-2 OS	–	Cep63 (C)	SAS6 (P)	Pericentrin (M)
DS5	RPE-1::tp53-/-	Centrinone	Cep63 (C)	Centrin-2 (C, P)	Pericentrin (M)

Only markers for centriole (C), procentriole (P) and pericentriolar matrix (M) are listed, as nuclei were stained with the DNA dye Hoechst in each case and were tabulated in Channel 0

### Model training

We trained the model without changing the hyperparameters on the training part of the dataset (Additional file 1: Fig. S2), keeping the remaining for evaluation. In addition, for both training and test sets, we used only one channel per field of view to prevent information from two similar channels of the same field of view to be present. To increase the variability of the training data, we used the following data augmentations: random affine transformation, random flip, random brightness-contrast and random gamma, as implemented in the image transformation suite Albumentations [9]. We trained the model for 100 epochs with binary cross-entropy error loss, the Adam optimiser (learning rate of 0.0003), a ReLU activation and a last activation of sigmoid type. As centriole/procentriole/PCM are sparse objects, we weighted the loss towards spots at the beginning of the training to prevent convergence to a black image, letting it decay towards the end of the training lest it induce false positives. All hyperparameters have been tuned during a preliminary phase using a small sample of the data and were not tuned afterwards.

### Model evaluation

To evaluate detection accuracy, we used the Hungarian algorithm to create unique matches between predictions and ground truth spots; matches were considered as true positive if the Euclidean distance between prediction and ground truth was below a given tolerance, namely 3 pixels (corresponding to ~ 300 nm). The resulting joint *CenFind* model reaches an average F_1_-score above 90% on the test set (Table 2). To further assess the robustness of the detection across immunofluorescence channels, we plotted the F_1_-score of each dataset as a function of the tolerance (i.e., the distance above which two entities are deemed to be distinct), which was varied from 0 to 5 pixels, finding the F_1_-score to be almost always maximal already at ~ 300 nm (Fig. 1i–m).

**Table 2:** Model comparisons

Method	F_1_-score on test set (mean ± st. dev.)
*CenFind* (Multiscale U-Net)	0.908 ± 0.043
U-Net	0.868 ± 0.073
Laplacian of Gaussian (LoG)	0.81 ± 0.230
OpenCV's Simple Blob Detector	0.615 ± 0.199
*FociDetector*	0.586 ± 0.254

Comparison of F_1_-scores (mean ± standard deviation) obtained on the test set (N = 15 fields of view) using the model of *CenFind* (Multiscale U-Net), the same model ablated in its multiscale (U-Net), the Laplacian of Gaussians (LoG), the OpenCV Simple Blob Detector, the *FociDetector* model from (4). Note that *FociDetector* uses a series of convolutions to highlight regions likely to have centrosomes and multiply them with the input, to compensate for the loss in resolution generated by the convolutions. Overall this results in a relatively high precision (0.852 ± 0.165), but in a substantially lower recall (0.519 ± 0.311)

### Comparison with existing methods

Several existing methods convolve a kernel over an image and score patches as containing a bright blob [10–12]. When the image of interest is convolved, one can extract the predicted blob by setting a threshold above which pixels are considered as being part of a blob. For comparison, we chose the implementation of scikit-image package that provides a functional API for Laplacian of Gaussians (LoG) and its derivatives [13, 14]. Here, the image is convolved with kernels of 10 different scales (σ) from 1 up to 2, which corresponds to blobs of size 1.4–2.8 pixels in radius. In addition, we ran the simple blob detector from the OpenCV library [15]; in this case, we converted the 16-bit image into 8-bit, as required by this method. We filtered the detected blobs by area between 5 and 100 pixels, leaving the brightness range from 0 to 255. We applied these two methods on our test set and computed the resulting F_1_-scores (Table 2). Although the conventional blob detection method LoG achieves satisfactory performances (average F_1_-score 0.810 ± 0.230), it performs substantially less well than *CenFind* (average F_1_-score 0.908 ± 0.043). Moreover, this is usually accompanied by a larger variance across channels, highlighting a lack of robustness reflected by a dramatic increase of false positives when the background is high.

Besides the above conventional computer-vision based approaches, we sought to also compare *CenFind* with an existing method that relies on convolutional neural network to detect centrosomes using a centriolar marker plus a PCM marker [4]. However, that pipeline requires a two channel input, a methodological difference that makes a direct comparison with *CenFind* difficult without substantial transformation of the code. We nevertheless extracted the sub-model *FociDetector*, which accepts a single channel image and passes it through four 5 × 5 convolution layers with 10 filters, each followed by a ReLU activation layer. We trained *FociDetector* for 100 epochs using the binary cross entropy as loss function, the stochastic gradient descent as optimiser, with a learning rate of 0.01, a momentum of 0.9 and a weigh decay of 0.0001. The resulting probability map was then multiplied with the original input mapped in the unit interval. Finally, the density map was analysed with the suite of functions implemented in *CenFind* and *SpotNet*. The model trained in this manner resulted in a F1-score of 0.586 ± 0.254 across the test set (Table 2), which is lower than that obtained with *CenFind*, although this may reflect the fact that the necessary code transformation may have impaired optimality.

## Materials and methods

### Dataset description

All datasets were z-stacks of unsynchronized human tissue culture cells immunostained for three distinct centriole/procentriole/PCM markers and stained with a DNA dye (see Table 1, Additional file 1: Tables S1 and S2 for specifics). Each dataset consisted of 24 or 25 fields of view containing on average 31 ± 14 cells per field of view; the fields of view were stored in the OME-TIFF format. We acquired three datasets (DS1–3) of retinal pigment epithelial cells (hTERT RPE-1; ATCC—CRL-4000, Batch #64,139,213; hereafter RPE-1) immunostained as reported in Table 1 and Additional file 1: Table S1. In addition, we acquired a dataset with osteosarcoma cells (U-2 OS; ECCAC, Merck—Cat# 92,022,711, Lot #10K035) (DS4). One further dataset was acquired with hTERT RPE-1::*tp53*^−/−^ cells in which procentriole formation was blocked with Centrinone (DS5) [16, 17].

### Cell culture, staining and imaging

RPE-1 and RPE-1::*tp*53^−/−^ cells were cultured in DMEM/F-12 (Thermo Fisher Scientific) with 10 % FBS (Merck), 0.2 mM sodium pyruvate (Thermo Fisher Scientific), and MEM non-essential amino acids (Thermo Fisher Scientific). U-2 OS cells were cultured in DMEM (Thermo Fisher Scientific) with 10 % FBS (Merck). Centrinone (MCE Cat# HY-18682) was used at a final concentration of 250 nM for 48 h.

For sample preparation, cells were grown on 15 mm glass coverslips (#1.5) and fixed with methanol at − 20 °C for 7 min, then permeabilized using PBS-T [0.05 % (v/v) Tween 20], before blocking for 1 h in PBS-T supplemented with 3 % BSA. All antibodies were diluted in blocking solution and incubated for 1 h at room temperature. Primary antibodies were mouse anti-Centrin-2 (Millipore Cat# 04–1624), rabbit anti-Cep63 (Millipore Cat# 06–1292), rabbit anti-Cep152 (Sigma-Aldrich Car# HPA039408), rabbit anti-CP110 (Bethyl Labs Cat# A301-344A), goat anti-Pericentrin (Santa Cruz Biotechnology Cat# sc-28143), mouse anti-SAS6 (Santa Cruz Biotechnology Cat# sc-81431), or mouse anti-γ-Tubulin (Sigma-Aldrich Cat# T6557). All primary antibodies were diluted 1000-fold, except Centrin-2 and Cep63, which were diluted 2000-fold. Secondary antibodies, used at 1000-fold dilution, were donkey anti-rabbit Alexa Fluor 488 (Thermo Fisher Scientific Cat# A21206), donkey anti-Mouse Alexa Fluor 568 (Thermo Fisher Scientific Cat# A10037), donkey anti-goat Alexa Fluor 647 (Thermo Fisher Scientific Cat# A21447). Following each antibody incubation, coverslips were washed three times with PBS-T and further incubated with 1 μg/mL Hoechst 33,258 (Sigma-Aldrich) in PBS before mounting in Fluoromount-G (Thermo Fisher Scientific).

Imaging was carried out on a Zeiss Observer D1 wide-field microscope with a 63 × oil immersion objective (NA 1.40) equipped with an Andor Zyla 4.2p camera. Z-sections were imaged at an interval of 300 nm. Images with a lateral resolution of 2048 pixels and a pixel size of 102.5 nm were saved as 16-bit OME TIFF files.

### Labelling workflow

The datasets were max-projected, then split into contrast-adjusted single channel images and manually annotated with the Labelbox online platform [18]. Foci of immunodetected proteins were marked with their X-Y coordinates (origin at the top left of the images) and downloaded as.csv files. Nuclei were segmented with a mixture of automated labelling using freehand box drawing and refined if necessary using freehand segmentation, and saved as masks.

### Software stack

The *CenFind* pipeline is implemented in Python version 3.9.5. Dependency management and packaging were implemented using Poetry and are available from pypi.org for download (see tutorial below on how to set up and use *CenFind*). The models were implemented in TensorFlow 2.0 [19]. Nuclei were segmented with *StarDist* using the DNA channel max-projected and binned so that the *StarDist* input is 256 × 256 pixels [6].

## Conclusions

Currently, centriole and procentriole numbers per cell in immunofluorescence experiments are usually determined manually by the experimenter, which represents a significant bottleneck towards reproducible and high-throughput data collection. Spot detection using classical computer vision cannot be used robustly with such a data modality due to the high likelihood of spurious blob detection. So far, the only deep learning pipeline designed in the field is focused on the detection of centrosome, not centrioles and procentrioles [4]. Furthermore, that pipeline relies on multichannel input. To address this methodological gap, we designed *CenFind*, which provides a channel-intrinsic, accurate and reproducible command line interface that automates centriole/procentriole detection across experimental modalities. The multi-scale convolution neural network core of *CenFind* allows the accurate detection of minute foci in high resolution images with sparse input. Moreover, the modular nature of *CenFind* enables its integration in larger pipelines via its API. By isolating the detection of centrioles/procentriole from the rest of the analysis, we anticipate that *CenFind* will find its place in broader analysis pipelines and prove critical for accelerating discoveries in the field. Of note, we provide a user guide to ensure adoption of *CenFind* by a broad community of scientists and to enable the pipeline to evolve for related detection tasks (Additional file 2).

Although an improvement over prior practice, *CenFind* is not without limitations. First, the pipeline uses a nuclear marker as a proxy to detect cells, which may be imperfect in some experimental conditions. Second, as with any deep-learning model, if new data to be analysed differs too much from the training data, the trained model is bound to underperform, although further training with the divergent dataset is expected to correct this.

### Availability and requirements

Project name: *CenFind*, Project home page: https://github.com/UPGON/cenfind, Operating system: Platform independent, Programming language: Python, Other requirements: Python 3.9.5 or higher, Licence: MIT.

## Supplementary Information

**Additional file 1: Table S1.** Number of nuclei in each dataset. Each dataset contains 24 or 25 fields of view. All nuclei were segmented, whether fully or partially visible (“Nuclei segmented”), before removal of partially visible nuclei, yielding "Nuclei analysed”, for determination of centriole/procentriole copy number per fully visible nucleus. **Table S2.** F1-scores of nuclear detections using *StarDist*. The centres of the annotated nuclei were compared to the predicted ones and the F1-score was computed. The tolerance was set to 50 pixels (5 µm). **Fig. S1.** Architecture of the *SpotNet* model. **a** *SpotNet*, the model used to detect centrioles/procentrioles/PCM, has a *U-Net* backbone that consists of an encoder, followed by a bottleneck and a decoder. The model takes as input a 2D image and is trained to detect centrioles/procentrioles/PCM foci at multiple interpolated versions of the image. The probability map generated by the model is passed to a local peak detection step, which converts it to a list of predicted foci. **b** Illustration of the comparison of predictions and annotations. **Fig. S2.** Ablation experiments with variations in depth of the *U-Net* backbone, spread of the spots in the ground truth masks and loss function. **a** *SpotNet* with loss = binary cross entropy depth = 3, spread = 1.5 (master model of *CenFind*). **b** *SpotNet* with varied depth: loss = binary cross entropy, depth = 2, σ = 1.5. **c** *SpotNet* with varied spread : loss = binary cross entropy, depth = 3, spread = 5. **d** *SpotNet* with varied loss: loss = mean absolute error, depth = 3, σ = 1.5. In each panel, the x axis represents the epoch, the y axis the loss (left) and the validation accuracy on the test data. (right)

**Additional file 2.** User guide for CenFind model improvement.

## Data Availability

The datasets generated and/or analysed during the current study are available in the Figshare repository https://figshare.com/articles/dataset/Cenfind_datasets/21581358; the model weights used for the performances are available in the Figshare repository https://figshare.com/articles/software/Cenfind_model_weights/21724421.

## Abbreviation

PCM: Peri-centriolar matrix

## References

[1] Gönczy P, Hatzopoulos GN (2019). Centriole assembly at a glance. J Cell Sci.

[2] Ryniawec JM, Rogers GC (2021). Centrosome instability: when good centrosomes go bad. Cell Mol Life Sci CMLS.

[3] Marteil G, Guerrero A, Vieira AF, de Almeida BP, Machado P, Mendonça S (2018). Over-elongation of centrioles in cancer promotes centriole amplification and chromosome missegregation. Nat Commun.

[4] Sankaran DG, Stemm-Wolf AJ, McCurdy BL, Hariharan B, Pearson CG (2020). A semi-automated machine learning-aided approach to quantitative analysis of centrosomes and microtubule organization. J Cell Sci.

[5] Dominges Mantes A, Herrera A, Khven I, Schläppi A, La Manno G, Weigert M. Spotipy: accurate and efficient spot detection for spatial transcriptomics. 2022. (in preparation). Available from: https://github.com/maweigert/spotipy.

[6] Schmidt U, Weigert M, Broaddus C, Myers G, Frangi AF, Schnabel JA, Davatzikos C, Alberola-López C, Fichtinger G (2018). Cell detection with star-convex polygons. Medical image computing and computer assisted intervention – MICCAI 2018.

[7] Weigert M, Schmidt U, Boothe T, Müller A, Dibrov A, Jain A (2018). Content-aware image restoration: pushing the limits of fluorescence microscopy. Nat Methods.

[8] He K, Zhang X, Ren S, Sun J. Delving deep into rectifiers: surpassing human-level performance on ImageNet classification [Internet]. arXiv; 2015 [cited 2023 Feb 12]. Available from: http://arxiv.org/abs/1502.01852

[9] Buslaev A, Iglovikov VI, Khvedchenya E, Parinov A, Druzhinin M, Kalinin AA (2020). Albumentations: fast and flexible image augmentations. Information.

[10] Xu Y, Wu T, Gao F, Charlton JR, Bennett KM (2020). Improved small blob detection in 3D images using jointly constrained deep learning and Hessian analysis. Sci Rep.

[11] Lindeberg T (1993). Detecting salient blob-like image structures and their scales with a scale-space primal sketch: a method for focus-of-attention. Int J Comput Vis.

[12] Meijering E, Dzyubachyk O, Smal I, van Cappellen WA (2009). Tracking in cell and developmental biology. Semin Cell Dev Biol.

[13] Marr D, Hildreth E (1980). Theory of edge detection. Proc R Soc Lond B Biol Sci.

[14] van der Walt S, Schönberger JL, Nunez-Iglesias J, Boulogne F, Warner JD, Yager N (2014). scikit-image: image processing in Python. PeerJ.

[15] Bradski G (2000). The openCV library. Dr Dobbs J Softw Tools Prof Program.

[16] Wang WJ, Acehan D, Kao CH, Jane WN, Uryu K, Tsou MFB (2015). De novo centriole formation in human cells is error-prone and does not require SAS-6 self-assembly. Elife.

[17] Wong YL, Anzola JV, Davis RL, Yoon M, Motamedi A, Kroll A (2015). Reversible centriole depletion with an inhibitor of Polo-like kinase 4. Science.

[18] Labelbox [Internet]. 2022 [cited 2022 Sep 5]. Available from: https://labelbox.com/education/.

[19] Abadi M, Agarwal A, Barham P, Brevdo E, Chen Z, Citro C, et al. TensorFlow: large-scale machine learning on heterogeneous distributed systems [Internet]. 2015 [cited 2022 Sep 5]. Available from: http://download.tensorflow.org/paper/whitepaper2015.pdf.

