# Supplementary material for: CenFind: a deep-learning pipeline for efficient centriole detection in microscopy datasets
Source: BMC Bioinformatics. 2023 Mar 28;24:120. doi: 10.1186/s12859-023-05214-2 (PMC10045196; doi:10.1186/s12859-023-05214-2)
Supplement: Supplementary file 1 — Additional file 1: Table S1. Number of nuclei in each dataset. Each dataset contains 24 or 25 fields of view. All nuclei were segmented, whether fully or partially visible (“Nuclei segmented”), before removal of partially visible nuclei, yielding "Nuclei analysed”, for determination of centriole/procentriole copy number per fully visible nucleus. Table S2. F1-scores of nuclear detections using StarDist. The centres of the annotated nuclei were compared to the predicted ones and the F1-score was computed. The tolerance was set to 50 pixels (5 µm). Fig. S1. Architecture of the SpotNet model. a SpotNet, the model used to detect centrioles/procentrioles/PCM, has a U-Net backbone that consists of an encoder, followed by a bottleneck and a decoder. The model takes as input a 2D image and is trained to detect centrioles/procentrioles/PCM foci at multiple interpolated versions of the image. The probability map generated by the model is passed to a local peak detection step, which converts it to a list of predicted foci. b Illustration of the comparison of predictions and annotations. Fig. S2. Ablation experiments with variations in depth of the U-Net backbone, spread of the spots in the ground truth masks and loss function. a SpotNet with loss = binary cross entropy depth = 3, spread = 1.5 (master model of CenFind). b SpotNet with varied depth: loss = binary cross entropy, depth = 2, σ = 1.5. c SpotNet with varied spread : loss = binary cross entropy, depth = 3, spread = 5. d SpotNet with varied loss: loss = mean absolute error, depth = 3, σ = 1.5. In each panel, the x axis represents the epoch, the y axis the loss (left) and the validation accuracy on the test data. (right) [file 12859_2023_5214_MOESM1_ESM.docx]

# Supplemental Data

| **Dataset** | **Nuclei segmented** | **Nuclei analysed** |
| --- | --- | --- |
| **DS1** | 659 | 518 |
| **DS2** | 635 | 515 |
| **DS3** | 668 | 521 |
| **DS4** | 358 | 276 |
| **DS5** | 1103 | 923 |

**Supplemental Table 1. Number of nuclei in each dataset.** Each dataset contains 24 or 25 fields of view. All nuclei were segmented, whether fully or partially visible (“Nuclei segmented”), before removal of partially visible nuclei, yielding "Nuclei analysed”, for determination of centriole/procentriole copy number per fully visible nucleus.

| **Dataset** | **F1** |
| --- | --- |
| **DS1** | 0.919 |
| **DS2** | 0.942 |
| **DS3** | 0.951 |
| **DS4** | 0.955 |
| **DS5** | 0.958 |

**Supplemental Table 2. F_1_-scores of nuclear detections using *StarDist*.** The centres of the annotated nuclei were compared to the predicted ones and the F_1_-score was computed. The tolerance was set to 50 pixels (5 µm).

**
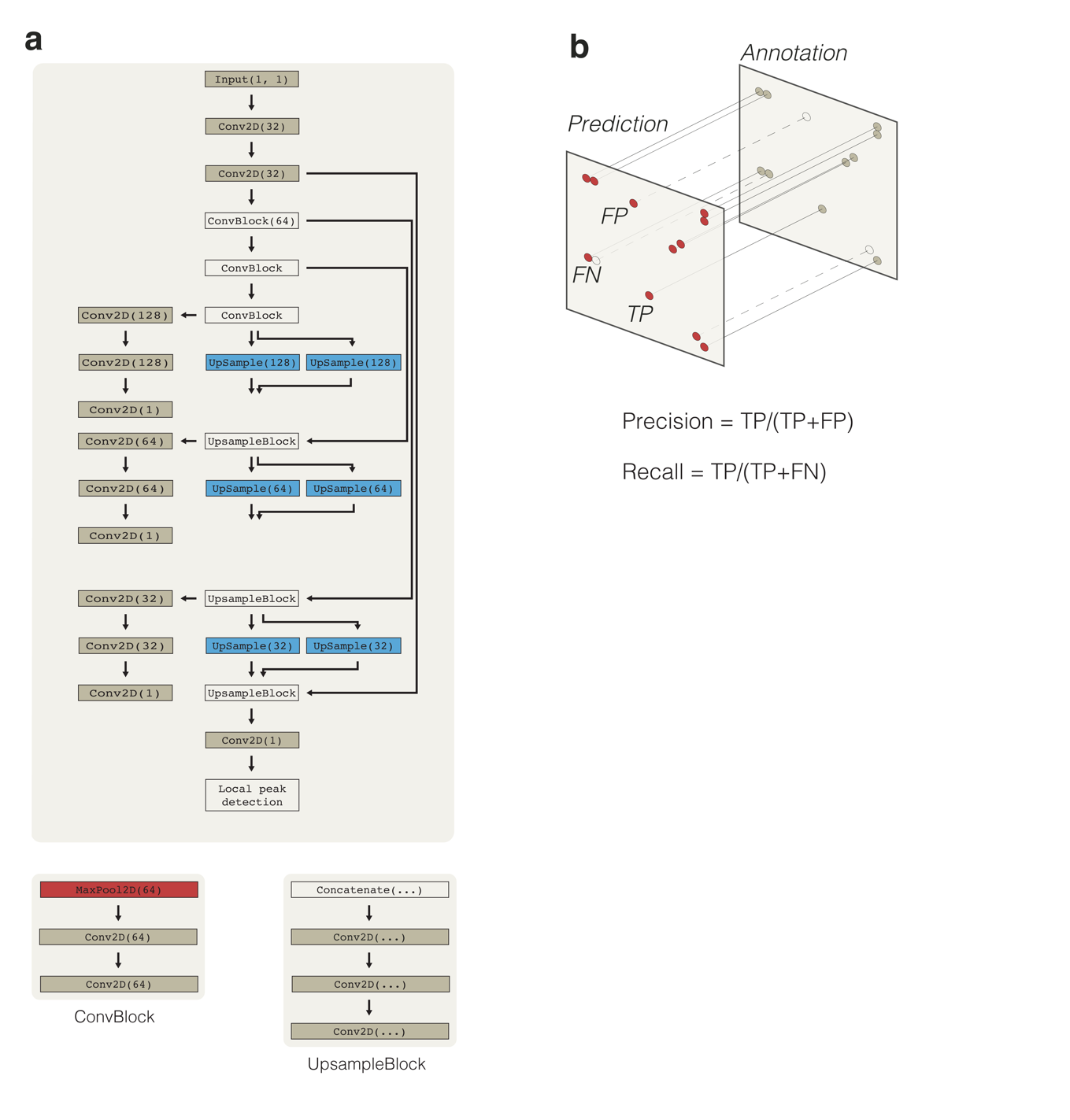
**

**Supplemental Figure 1. Architecture of the *SpotNet* model. a.** *SpotNet*, the model used to detect centrioles/procentrioles/PCM, has a *U-Net* backbone that consists of an encoder, followed by a bottleneck and a decoder. The model takes as input a 2D image and is trained to detect centrioles/procentrioles/PCM foci at multiple interpolated versions of the image. The probability map generated by the model is passed to a local peak detection step, which converts it to a list of predicted foci. **b**. Illustration of the comparison of predictions and annotations.

**a**
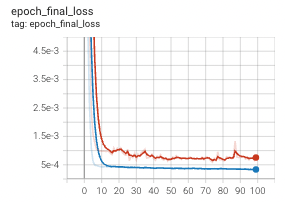

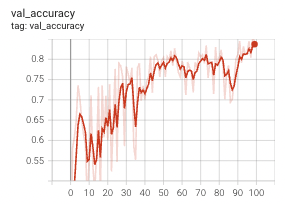


**b**


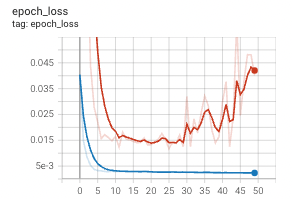

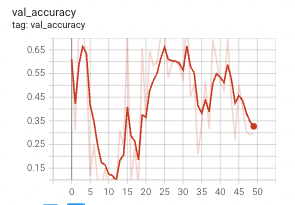


**c**


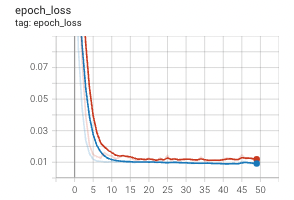

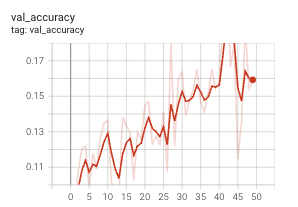


**d**


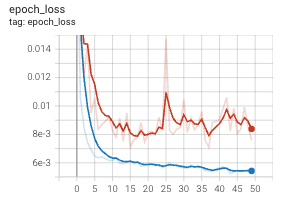

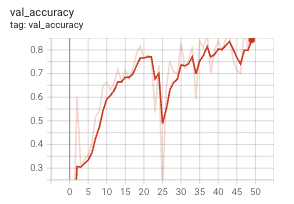


**Supplemental Figure 2. Ablation experiments with variations in depth of the *U-Net* backbone, spread of the spots in the ground truth masks and loss function.** **a**. *SpotNet* with loss = binary cross entropy depth = 3, spread = 1.5 (master model of *CenFind*). **b**. *SpotNet* with varied depth: loss = binary cross entropy, depth = 2, 𝜎 = 1.5. **c**. *SpotNet* with varied spread : loss = binary cross entropy, depth = 3, spread = 5. **d**. *SpotNet* with varied loss: loss = mean absolute error, depth = 3, 𝜎 = 1.5. In each panel, the x axis represents the epoch, the y axis the loss (left) and the validation accuracy on the test data. (right).
