# Supplementary material for: CenFind: a deep-learning pipeline for efficient centriole detection in microscopy datasets
Source: BMC Bioinformatics. 2023 Mar 28;24:120. doi: 10.1186/s12859-023-05214-2 (PMC10045196; doi:10.1186/s12859-023-05214-2)

**CenFind: User guide for model improvement**

The initial model M is fitted using a set of five representative datasets, hereafter referred to as the *standard datasets* (DS1-5). If your type of data deviates too much from the standard dataset, M may perform less well. Specifically, when setting out to score a new dataset, you may be faced with one of three situations, as reflected by the corresponding F_1_ score (i.e., 2TP/2TP+FN+FP, TP: true positive, FP: false positive; FN: false negative): (1) the initial model (M) performs well on the new dataset (0.9 ≤ F_1_ ≤ 1); in this case, model M is used; (2) model M performs significantly worse on the new dataset (0.5 ≤ F_1_ < 0.9); in this case, you may want to consider retraining the model (see below); (3) the model does not work at all (0 ≤ F_1_ < 0.5); such a low F_1_value probably means that the features of the data set are too distant from the *standard datasets* to warrant retraining starting from M. Before retraining a model in case (2), verify once more the quality of the data, which needs to be sufficiently good in terms of signal over noise to enable efficient learning. If this is not the case, it is evident that the model will not be able to learn well. If you, as a human being, cannot tell the difference between a real focus and a stray spot using a single channel at hand (i.e., not looking at other channels), the same will hold for the model. To retrain the model, you first must annotate the dataset, divide it randomly, using the CenFind's utility *cenfind prepare,* to training and test sets (90 % versus 10 % of the data, respectively). Next, the model is trained with the 90 % set, thus generating a new model, M*. Last, you will evaluate the gain of performance on the new dataset, as well as the potential loss of performance on the *standard datasets*.

Detailed training procedure (see also figure below):

1. Split the dataset into training (90%) and test (10%) sets, consisting of pairs of one field of view and the channel to use. This helps trace back potential issues that may arise during the training and renders the model fitting reproducible.
   cenfind prepare [-h] [--projection_suffix PROJECTION_SUFFIX] [--splits SPLITS [SPLITS ...]] dataset
2. Label all the images present in the training and test sets using Labelbox.
3. Save all foci coordinates (x, y), origin at top-left, present in one field of view as one text file under /path/to/dataset/annotation/centrioles/ with the naming scheme <dataset_name>_max_C<channel_index>.txt.
4. Evaluate the newly annotated dataset using the model M by computing the F_1_ score.
   cenfind evaluate [-h] [--performances_file PERFORMANCES_FILE] [--tolerance TOLERANCE] --channel_nuclei CHANNEL_NUCLEI --channel_centrioles CHANNEL_CENTRIOLES [CHANNEL_CENTRIOLES ...] [--vicinity VICINITY] dataset model
5. If the performance is poor (i.e., F_1_ score < 0.9), fit a new model instance, M*, with the standard dataset plus the new dataset (90% in each case).
6. Test performance of model M* on the new data set; hopefully the F_1_ score will now be ≥ 0.9 (if not: consider increasing size of annotated data).
7. Test performance of model M* on the standard datasets; if performance of F_1_* ≥ F_1_, then save M* as the new M (otherwise keep M* as a separate model for the new type of data set).


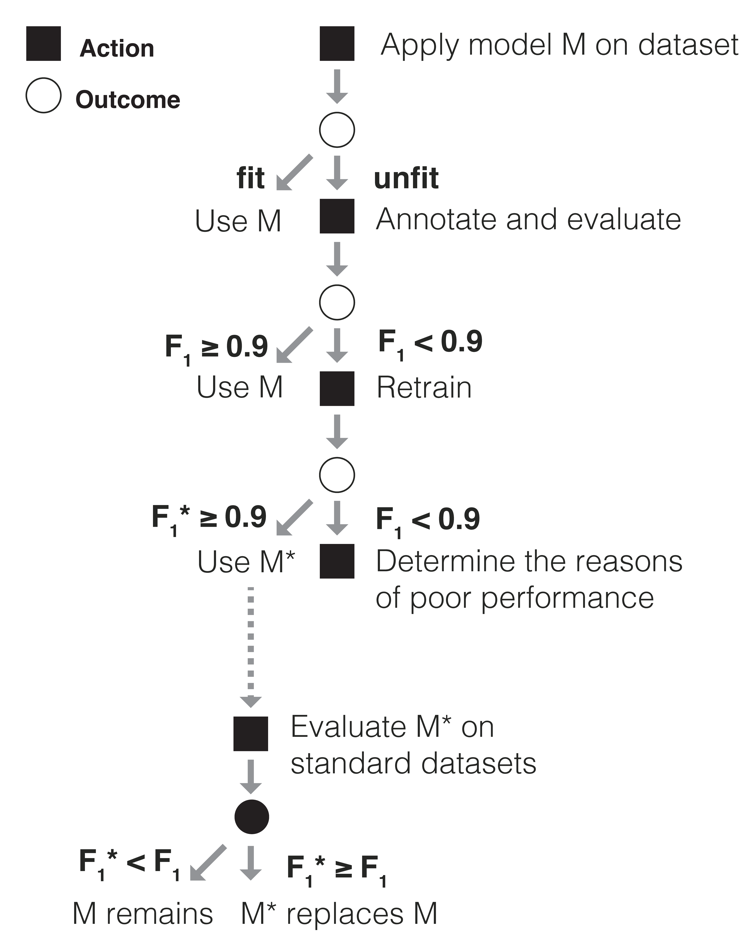

Supplement: Supplementary file 2 — Additional file 2. User guide for CenFind model improvement. [file 12859_2023_5214_MOESM2_ESM.docx]
